# Performance of case definitions and clinical predictors for influenza surveillance among patients followed in a rural cohort in Senegal

**DOI:** 10.1186/s12879-020-05724-x

**Published:** 2021-01-07

**Authors:** Mamadou Aliou Barry, Florent Arinal, Cheikh Talla, Boris Gildas Hedible, Fatoumata Diene Sarr, Ibrahim Oumar Ba, Boly Diop, Ndongo Dia, Muriel Vray

**Affiliations:** 1 Institut Pasteur de Dakar, Unité d’Epidémiologie des maladies infectieuses, 36, Avenue Pasteur, Dakar, Sénégal; 2 Organisation Mondiale de la Santé-Dakar, Dakar, Sénégal; 3 Ministère de la Santé et de l’Action Sociale, Direction de la Prévention, Dakar, Sénégal; 4 Institut Pasteur de Dakar, Pôle de Virologie, Dakar, Sénégal

**Keywords:** Performance, Influenza, Surveillance, Senegal

## Abstract

**Background:**

Influenza is a major cause of morbidity and mortality in Africa. However, a lack of epidemiological data remains for this pathology, and the performances of the influenza-like illness (ILI) case definitions used for sentinel surveillance have never been evaluated in Senegal. This study aimed to i) assess the performance of three different ILI case definitions, adopted by the WHO, USA-CDC (CDC) and European-CDC (ECDC) and ii) identify clinical factors associated with a positive diagnosis for Influenza in order to develop an algorithm fitted for the Senegalese context.

**Methods:**

All 657 patients with a febrile pathological episode (FPE) between January 2013 and December 2016 were followed in a cohort study in two rural villages in Senegal, accounting for 1653 FPE observations with nasopharyngeal sampling and influenza virus screening by rRT-PCR. For each FPE, general characteristics and clinical signs presented by patients were collected. Sensitivity, Specificity, Positive Predictive Value (PPV) and Negative Predictive Value (NPV) for the three ILI case definitions were assessed using PCR result as the reference test. Associations between clinical signs and influenza infection were analyzed using logistic regression with generalized estimating equations. Sore throat, arthralgia or myalgia were missing for children under 5 years.

**Results:**

WHO, CDC and ECDC case definitions had similar sensitivity (81.0%; 95%CI: 77.0–85.0) and NPV (91.0%; 95%CI: 89.0–93.1) while the WHO and CDC ILI case definitions had the highest specificity (52.0%; 95%CI: 49.1–54.5) and PPV (32.0%; 95%CI: 30.0–35.0). These performances varied by age groups. In children < 5 years, the significant predictors of influenza virus infection were cough and nasal discharge. In patients from 5 years, cough, nasal discharge, sore throat and asthenia grade 3 best predicted influenza infection. The addition of “nasal discharge” as a symptom to the WHO case definition decreased sensitivity but increased specificity, particularly in the pediatric population.

**Conclusion:**

In summary, all three definitions studies (WHO, ECDC & CDC) have similar performance, even by age group. The revised WHO ILI definition could be chosen for surveillance purposes for its simplicity. Symptomatic predictors of influenza virus infection vary according the age group.

**Supplementary Information:**

The online version contains supplementary material available at 10.1186/s12879-020-05724-x.

## Background

Acute respiratory infections (ARIs) are a major cause of morbidity and mortality in most African countries, especially in children under five years. In terms of ARI etiology, respiratory virus infections are very common, with influenza virus the most common [1–4]. Indeed, influenza contributes substantially to the morbidity and mortality of respiratory infections, and the highest burden of severe disease is experienced by the < 5 and ≥ 65 years age groups [5]*.* This vaccine preventable disease is characterized by seasonal epidemics that occur throughout the world every year, and occasional pandemics arising from novel subtypes. The World Health Organization (WHO) estimates that 3 to 5 million severe cases of influenza occur each year, among which 290,000 to 650,000 cases in the very young, elderly and patients with comorbidities [6]. Almost all influenza-associated deaths in children (99%) occur in developing countries [7].

The burden of influenza, and the gaps in understanding of influenza epidemiology in African countries, necessitate increasing surveillance of influenza. This is all the more pertinent following the emergence of avian influenza viruses and the latest influenza pandemic [8, 9].

Currently, community-level surveillance depends on influenza-like illness (ILI) as severe acute respiratory infections (SARI) are a common feature of influenza surveillance but focuses on hospitalized cases. WHO has developed and disseminated standardized procedures, part of the Integrated Disease Surveillance and Response Strategy (IDSR), and proposes a simple, easily understandable and easily implemented definition of ILI for surveillance of Influenza. Since 2014, the WHO case definition for ILI is “any measured temperature greater than or equal to 38°C and cough, with onset within the last 10 days “ [10–12].

However, the international standardization of this definition is difficult, because the optimal choice of case definition depends on population studied and the surveillance objectives. Other case definitions include, in addition to fever and cough, symptoms such as arthralgia, myalgia or headache. Further complication in the standardization of an international case definition derives from the fact that clinical symptoms of influenza are often nonspecific and not easily distinguishable from other infectious etiologies in patients with acute febrile illness [13].

Many studies have compared the overall performances of case definitions recommended for influenza surveillance by WHO, the United States Centers for Disease Prevention and Control (CDC), and the European Center for Disease Control and Prevention (ECDC). However, the majority of these studies were focused on populations in developed countries, especially in hospitals. In resource-limited settings, data or knowledge about the performances of ILI case definitions is still very limited. Diagnosis in children under five remains difficult because of a clinical presentation often different from the symptoms included in ILI’s case definitions [14–20].

Based on data collected in a cohort-based study conducted in a Senegalese rural area, we aimed i) to compare the performances of international clinical case definitions used by WHO, CDC, and ECDC for the diagnosis of influenza ii) to identify clinical factors associated with a positive diagnosis of influenza viruses and iii) to propose a diagnostic algorithm for the Senegalese context.

## Methods

### Study design and setting

#### Presentation of the Syndromic sentinel surveillance network in Senegal (4S network)

Senegal, a sub-Saharan Africa country, has a long-standing influenza surveillance system, initially focused on virological surveillance from 1996 and then adopted a syndromic approach since 2011 through the establishment of the Senegalese Syndromic Sentinel Surveillance Network (4S network) from a partnership between Ministry of Health (MoH), the WHO country office and the Institut Pasteur of Dakar (IPD) which hosts the National Influenza Centre [21]. In addition to influenza-like illness (ILI), other public health priority syndromes (malaria, dengue-like syndromes and diarrheal syndromes) have been added through an integrated approach. An early warning system (EWS) of diseases under surveillance was set up in 2015 that allows the MoH to detect and alert quickly any abnormal health event. The 4S network provides ‘real time’ data on influenza epidemiology, seasonality and also circulating strains over the country.

#### Sentinel sites

The 4S sites were selected on the basis of criteria used by the MoH. A checklist criteria was developed based on the WHO recommended attributes for sentinel site selection including, feasibility, representativeness and the availability of data to enable disease burden estimate (Additional file 1). Of the 20 ILI surveillance sentinel sites in the 4S network, two sites in Dielmo and Ndiop were chosen for this study.

### Study population

**Fig. 1:**
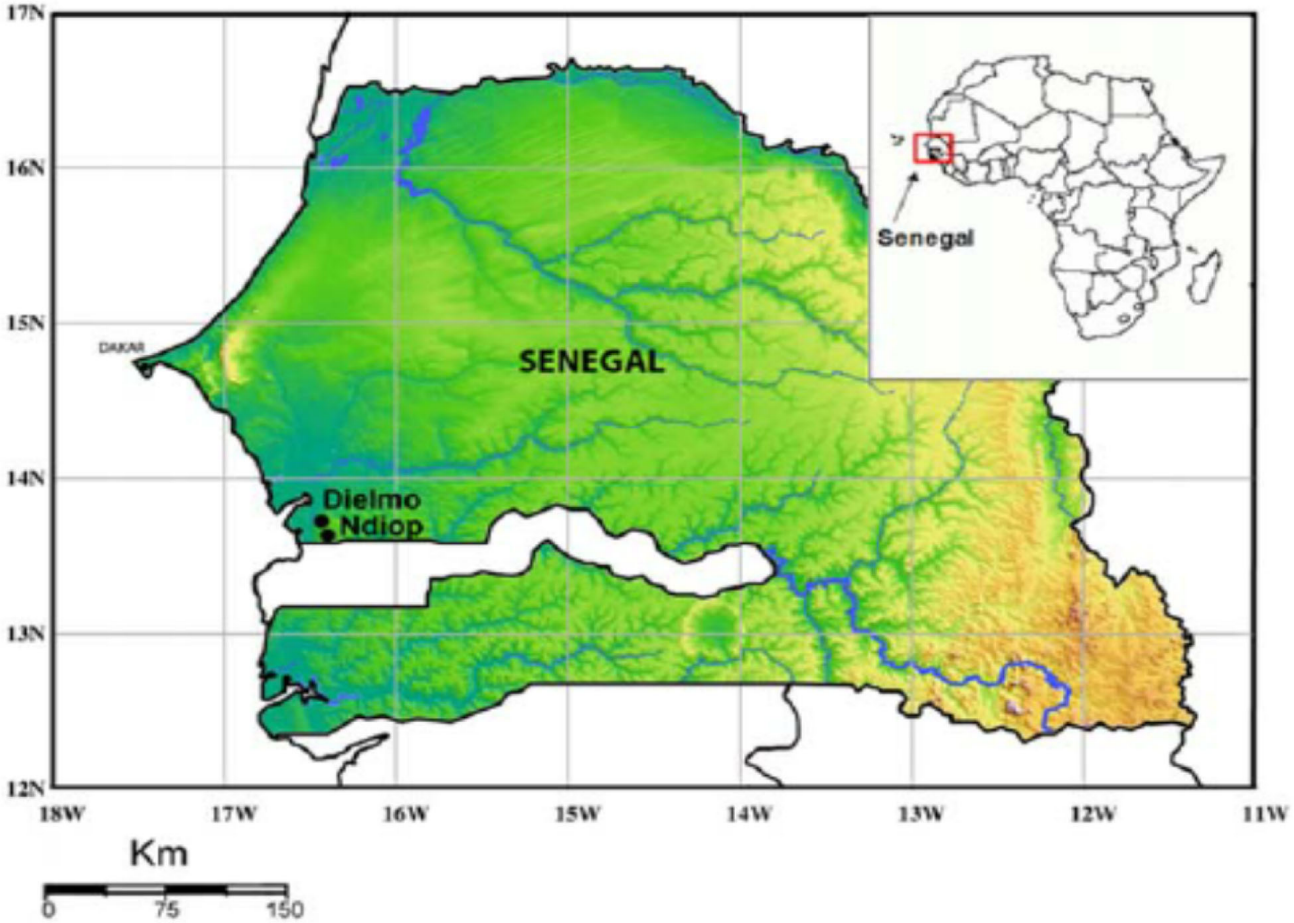
Location of Dielmo and Ndiop villages in Senegal, WestAfrica.doi:10.1371/journal.pntd.0001999.g001

Dielmo and Ndiop are two villages in a Sudanese region in southwestern Senegal located in the district of Sokone (Fig. 1). All inhabitants in the two villages have been included in a malaria cohort study for over 25 years [22, 23]. As part of the strategy for eliminating malaria in Africa, anyone living in the study area and having experienced a febrile episode systematically benefits from a blood sample for malaria research. Faced with the persistence of febrile syndromes despite a decrease in the incidence of malaria in this area, the strategy of systematic collection and testing of swabs from individuals with fever, not only from patients with ILI, particularly free from malaria, was adopted in order to assess the burden of the influenza virus in rural areas.

For the current study, if anyone in the village experienced a Febrile Pathological Episode (FPE), they presented to the health center, spontaneously or after an active screening conducted daily in all households. All patients with fever, defined by a corrected axillary temperature ≥ 38 °C at the time of the clinical examination evolving for less than 10 days, had a nasopharyngeal swab taken, and their clinical symptoms recorded. If one participant experienced two FPEs within a period of more than 15 days, the FPEs were considered as separate events.

### Data collected

#### Clinical data

The data collected included general characteristics (age, gender, place of residence) and clinical symptoms, defined by presence/absence (fever, cough, sore throat, headache, asthenia, myalgia, arthralgia, abdominal pain, anorexia, diarrhea, nausea/vomiting, dyspnea, sweats, chills). Asthenia was further defined in 3 grades as follow: grade 1 (presence of unusual fatigue); grade 2 (fatigue that impacts daily activities) and grade 3 (fatigue that does not allow the patient to leave his bed). Sore throat, arthralgia, myalgia or headache were not or less reported in children under 5 years.

The definitions used for influenza-like illness (ILI) surveillance by WHO, CDC and ECDC are summarized in Table 1.

**Table 1:** Cases definitions, three international ILI definitions, Dielmo and Ndiop cohort study, Senegal, 2013–2016

Organisation	Clinical symptoms
WHO*	Sudden onset AND Measured fever of ≥ 38 °C AND Cough With onset within the last 10 days
CDC^¶^	Sudden onset AND Fever ≥ 100 ° F (37.8 °C) Absence of a known cause other than influenza AND At least one among: cough, sore throat
ECDC”	Sudden onset AND At least one among: fever, feverishness, headache, malaise, myalgia AND At least one among: cough, sore throat, shortness of breath

WHO* World Health Organization, *http://www.who.int/influenza/resources/documents/**technical_consultation/en/;*

*CDC*^¶^ Centers for Disease Control and Prevention; U.S. *Outpatient Influenza-like Illness Surveillance Network (ILINet)*

*ECDC*” *European Centre for Disease Prevention and Control;* *https://www.ecdc.europa.eu/sites/default/files/documents/influenza*

#### Laboratory data

For all febrile patients, a nasopharyngeal sample was collected, placed in 2 mL of Universal Transport Medium (COPAN Diagnostics) and stored at 4 °C before being sent weekly to the National Influenza Center (NIC) of Senegal, Institute Pasteur of Dakar (IPD). For respiratory viruses, a two-step Real-Time Reverse Transcription–Polymerase Chain Reaction (RT-PCR) was performed using the CFX96 Real-time PCR system (Bio-Rad) and Anyplex II RV16 Detection kit (Seegene), as previously described [24].

### Statistical analysis

#### Description of the population

Characteristics of patients with one or more FPEs were described by sex and age. Continuous variables were summarized as means with standard deviation or as median with interquartile range (IQR); the student’s T test was used to compare the means. Dichotomous or categorical variables were described by percentages with 95% confidence interval and Chi-square test or Fisher’s exact test used to compare proportions between categories. Positivity rates for influenza were calculated by sex, age groups and clinical signs. Proportions missing for symptoms collected from children aged < 5 years were reported.

The sensitivity, specificity, Positive Predictive Value (PPV) and Negative Predictive Value (NPV) of each of the three ILI case definitions were assessed globally and by age groups (0 - < 2, 2 – < 5, 5 – < 15, 15 - < 50, ≥ 50 years) using PCR results as reference test. Sensitivity was defined as the proportion of subjects identified by the ILI case definitions among those with PCR positive result for influenza. The specificity was defined as the proportion of subjects not identified by the ILI case definitions among those with PCR negative result for influenza. The PPV was defined as the probability of having laboratory-confirmed influenza when the symptoms (ILI case definitions) are present. The NPV was defined as the probability of not having laboratory-confirmed influenza when the symptoms (ILI case definitions) are not present. The differences in sensitivity and specificity of the CDC and ECDC ILI definitions were calculated against the WHO definition and compared using Chi-square tests.

The analysis was not limited to the first FPE observation in each patient. As several FPEs could occur over time for a same subject, models with generalized estimating equations (GEE) were performed on longitudinal data to determine the impact of clinical variables on a positive influenza diagnostic [25]. The univariate and multivariate analyses were performed according two age groups (< 5 years & >= 5 years) to identify factors potentially associated with positive virological diagnosis for influenza. All variables potentially associated with laboratory-confirmed Influenza in univariate analysis with a *p*-value of < 0.25 were included in a backward multivariable logistic regression model to investigate the combined influence of clinical variables as potential independent predictive factors for laboratory-confirmed Influenza. A *p*-value below 0.05 (two-sided test) was considered statistically significant. Model validity was tested using the Wald test. Trend tests were performed on categorical variables at several levels.

The variables found in the final multivariable logistic regression model were then compared to the WHO ILI case definition. In children under 5 years, missing symptoms myalgia, sore throat and arthralgia were excluded in both univariate and multivariate analysis and proportions missing for headache were also excluded in the analysis (case-complete analysis method).

The statistical analysis was performed with Stata version15 software (StataCorp, LLC, College Station, TX, USA).

### Ethical considerations

The project was approved by the Senegalese National Ethics Committee of the Ministry of Health. Written informed consent was obtained from all participants or from legal guardians in case of minor patients, before inclusion in the study.

## Results

### General description of the population (Table 2)

Between January 1, 2013 and December 31, 2016 1653 FPEs were observed from 657 subjects. Of these 657 patients, 344 (52.4%) were female, 189 (29.0%) were children under 5 years and 251 (38.2%) presented with a single FPE. The median age of all patients was 10.4 years (IQR: 3.3–22.0 years). The comparison between subjects with single and multiple FPEs did not show any difference regarding the sex (*p* = 0.40). However, a significantly higher proportion of patients with more than one FPE was found in patients >= 5 years (265/406, 65.3%) compared to those under 5 years of age (*p*< 0.001).

**Table 2:** Description of the FPEs population, by sex, and age groups enrolled in the study, Dielmo and Ndiop, Senegal, 2013–2016

	Patients presenting with FPEs* and included in study, 2013–2016 (***n***=657)	Only one FPE*, 2013–2016 (***n***=251)	More than one FPE*, 2013–2016 (***n***=406)	***P***-values
	no (%)	no (%)	no (%)	
**Gender**				
Female	344 (52.4)	137 (54.6)	207 (51.0)	0.40
**Age group (years)**				
< 5	189 (29.0)	48 (19.0)	141 (34.7)	< 0.001
≥ 5	468 (71.2)	203 (81.0)	265 (65.3)	

FPEs* febrile pathologic episodes

### Confirmed influenza cases

Overall, 21.6% (357/1653) of the FPEs tested positive for influenza infection by RT- PCR. This proportion is higher for the year 2015 (37.0% versus 14.0, 18.0 and 15.0% for the years 2013, 2014 and 2016, respectively). A peak of influenza circulation was identified between weeks 33 and 49 of each year corresponding to the seasonality of influenza viruses in Senegal.

Among the 357 FPEs with positive PCR for influenza, 47.9% (171/357) were for influenza B and 52.1% (186/357) for influenza A. For influenza A cases, A *(H1N1) pdm09* and A*(H3N2)* viruses were detected in 19.3% (69/357) and 31.9% (114/ 357), respectively. The distribution of influenza A and B subtypes were not significantly different between age groups (*p* = 0.407). Influenza A *(H1N1) pdm09* and influenza B viruses predominated during the 2013 and 2015 seasons, while influenza A (*H3N2*) predominated during the 2014 and the 2016 seasons.

The proportion of FPEs associated with a positive PCR for influenza increased significantly with age: 14.6% (44/302), 22.0% (78/355), 23.0% (129/561), 23.2% (85/366) and 30.4% (21/69), respectively for 0- < 2 years, 2- < 5 years, 5- < 15 years, 15- < 50 years, > = 50 years (*p*-trend < 0.01).

### Clinical symptoms (Table 3)

The most common symptoms reported among the 1653 FPEs were headache (92.1%), cough (55.3%), asthenia grade 3 (55.0%), and nasal discharge (42.2%) with a significant difference between FPEs with influenza virus-positive PCR compared to those with influenza-negative PCR (*p*< 0.001; p< 0.001, *p*=0.07 and *p*< 0.0001, respectively). In children under 5 years, 657 FPEs (40.0%) were missing for each of the following symptoms sore throat, myalgia and arthralgia vs 356 FPEs (21.6%) missing for headache.

**Table 3:** FPEs with positive and negative PCR for influenza by sex, age groups, and clinical symptoms enrolled in the study, Dielmo and Ndiop, Senegal, 2013–2016

	All cases of FPEs (***n***=1653)	FPEs with positive PCR for influenza A or B (***n***=357)	FPEs with negative PCR for influenza A or B (***n***=1296)	***P***-values
	***n*** (%)	***n*** (%)	***n*** (%)	
**Gender**				
Female	827 (50.0)	181 (51.0)	646 (50.0)	0.77
**Age groups** (years)				
0 – < 2	302 (18.3)	44 (12.3)	258 (20.0)	0.01
2 - < 5	355 (21.4)	78 (22.0)	277 (21.3)	
5 - < 15	561 (34.0)	129 (36.1)	432 (33.3)	
15- < 50	366 (22.1)	85 (24.0)	281 (21.6)	
≥ 50	69 (4.2)	21 (6.0)	48 (4.0)	
*Mean age (SD°)*	*12.5 ± (14.6)*	*14.0 ± (15.3)*	*12.1 ± (14.5)*	
*Median age (IIQ¶)*	*7.1 ± (3.0–16.0)*	*8.1 (3.6–18.0)*	*7.0 (2.3–15.2)*	
**Clinical symptoms**				
Headache^**1**^	1184/1286 (92.1)	291/301 (96.6)	893/985 (90.6)	< 0.001
Cough	914 (55.3)	289 (81.0)	625 (48.2)	< 0.001
Asthenia grade 0	229 (14.0)	43 (12.0)	186 (14.3)	0.07
Asthenia grade 1	95 (6.0)	13 (3.6)	82 (6.3)	
Asthenia grade 2	424 (25.6)	87 (24.4)	337 (26.0)	
Asthenia grade 3	905 (55.0)	214 (60.0)	691 (53.3)	
Anorexia	759 (46.0)	180 (50.4)	579 (45.0)	0.05
Nasal discharge	697 (42.2)	254 (71.1)	443 (34.2)	< 0.0001
Myalgia^**1**^	604/992 (61.0)	132/235 (56.2)	472/757 (62.3)	0.18
Chills	416 (25.2)	86 (24.1)	330 (25.5)	0.60
Nausea/vomiting	326 (20.0)	51 (14.3)	275 (21.2)	< 0.01
Sore throat^**1**^	187/996 (19.0)	68/235 (29.0)	119/761 (15.6)	< 0.001
Sweats	171 (10.3)	35 (10.0)	136 (10.6)	0.71
Arthralgia^**1**^	157/996 (15.8)	37/209 (18.0)	120/787 (15.2)	0.52
Diarrhea	154 (9.3)	20 (5.6)	134 (10.3)	< 0.01
Abdominal pain	59 (3.6)	7 (2.0)	52 (4.0)	0.06
Dyspnea	47 (3.0)	19 (5.4)	28 (2.2)	< 0.01

*FPEs*^”^ febrile pathological episodes, *SD* Standard Deviation, *IIQ¶* interquartile range

^**1**^: These symptoms were not collected or were incomplete in children aged under 5 years

-Headache: 356 FPEs (21.5%) missing in children under 5 years

-Sore throat, Myalgia and Arthralgia symptoms: 657 FPEs (40.0%) missing in all children under 5 years for each of these 3 symptoms

### Case definitions performances (Table 4)

The performance of the three case definitions are shown in Table 4. Of the 1653 FPEs reported during the study period, 914 (55.3%), 915 (55.4%) and 932 (56.4%) met the WHO, CDC and ECDC ILI case definitions, respectively. Overall, the performances of the ILI case definitions used by WHO, CDC and ECDC were similar, globally and by age (*p*=0.990). However, the performances of these case definitions varied significantly between age groups. Sensitivity was above 72.0% for any age group, while the specificity varied from 36.0% among the 0-< 2 years-old to 58.0% among 5- < 15 years-old and above 65.0% in adult population (≥15 years). The corresponding positive predictive values ranged from 18% for 0- < 2 year to 50.0% for patients above 50 years. No differences were observed in negative predictive values between age groups (*p*> 0.05).

**Table 4:** Performance of WHO, CDC and ECDC case definitions tested for detection of influenza globally and by age groups, Dielmo and Ndiop study, Senegal, 2013–2016

Case definitions	Sensitivity % (95% CI)	Specificity % (95% CI)	PPV* % (95% CI)	NPV^†^ % (95% CI)
**All cases**				
WHO	81.0 (77.0–85.0)	52.0 (49.1–54.5)	32.0 (30.0–35.0)	91.0 (89.0–93.1)
CDC	81.0 (77.0–85.1)	52.0 (49.1–54.5)	32.0 (30.0–35.0)	91.0 (88.5–93.1)
ECDC	82.0 (77.5–85.5)	50.5 (48.0–53.2)	31.2 (28.2–34.2)	91.0 (89.0–93.1)
**Age groups (years)**				
**0 - < 2**				
WHO	82.0 (70.4–93.2)	36.0 (30.0–41.5)	18.0 (13.0 -- 23.3)	92.0 (87.0–97.3)
CDC	82.0 (70.4–93.2)	36.4 (30.6–42.3)	18.0 (12.5–23.3)	92.2 (86.0–97.4)
ECDC	84.1 (73.3–95.0)	36.0 (30.0–41.5)	18.2 (13.0–23.5)	93.0 (88.0–98.0)
**2 - < 5**				
WHO	86.0 (78.3–94.0)	38.0 (32.3–44.0)	28.0 (22.3–33.6)	90.5 (85.2–96.0)
CDC	86.0 (77.0–94.0)	38.2 (32.5–44.0)	28.2 (22.5–34.0)	90.6 (85.3–96.0)
ECDC	86.0 (78.2–94.0)	38.0 (31.2–43.6)	28.0 (22.3–34.0)	90.5 (85.1–96.0)
**5 - < 15**				
WHO	84.0 (77.3–90.1)	58.0 (53.2–62.5)	37.2 (31.6–42.7)	92.2 (89.0–95.4)
CDC	84.4 (78.2–91.0)	57.0 (52.3–61.6)	37.0 (31.5–42.5)	92.5 (89.3–95.6)
ECDC	84.5 (78.2–91.0)	56.0 (51.3–61.0)	36.5 (31.0–42.0)	92.3 (89.1–95.5)
**15 - < 50**				
WHO	72.0 (62.2–81.3)	69.0 (63.3–74.1)	41.0 (33.1–49.0)	89.0 (85.0–93.2)
CDC	72.0 (62.2–81.3)	69.0 (63.6–74.4)	41.2 (33.3–49.2)	89.0 (85.0–93.1)
ECDC	72.0 (62.2–81.3)	66.0 (60.3–71.4)	39.0 (31.4–46.6)	88.5 (84.2–93.0)
**≥ 50**				
WHO	81.0 (64.2–980)	65.3 (52.0–78.6)	50.0 (33.2–67.0)	88.5 (78.0–99.1)
CDC	81.0 (64.1–98.0)	65.0 (51.0–78.1)	50.0 (33.1–67.0)	88.6 (78.1–99.2)
ECDC	81.0 (64.1–98.0)	65.0 (51.1–78.1)	50.0 (33.2–67.0)	88.6 (78.1–99.1)

PPV*= Positive Predictive Value**; NPV**^†^**=** Negative Predictive Value

### Clinical predictors associated with positive PCR for influenza (Tables 5 & 6)

In univariate analysis, only cough, nasal discharge, headache, asthenia, diarrhea and dyspnea were found to be significantly associated with a positive PCR for influenza in all age group. The presence of sore throat, anorexia and nausea/vomiting were significantly associated with influenza in the age group >= 5 years.

**Table 5:** factors associated with laboratory-confirmed influenza in children under 5 years, Dielmo and Ndiop cohort study, Senegal, 2013–2016 (*n* = 657^1^)

Variable	Univariate analysis			Multivariate analysis		
	OR	95% CI	***P*** value	aOR¶	95% CI	***P*** value
**Sex**						
Women	1.12	[0.78–1.61]	0.53			
**Clinical symptoms**^**a**^						
Headache^b^	1.5	[1.00–2.32]	0.06			
Cough	3.15	[2.0–5.12]	< 0.0001	2.21	[1.30–3.85]	< 0.01
Asthenia grade 0	Ref.*					
Asthenia grade 1	0.35	[0.12–1.01]	0.05			
Asthenia grade 2	1.0	[0.52–1.63]	0.77			
Asthenia grade 3	0.84	[0.50–1.38]	0.50			
Anorexia	1.13	[0.80–1.64]	0.52			
Chills	0.80	[0.35–1.80]	0.57			
Nausea/Vomiting	0.75	[0.45–1.24]	0.26			
Sweats	0.77	[0.33–1.74]	0.60			
Diarrhea	0.65	[0.30–1.35]	0.24			
Nasal discharge	2.82	[1.81–4.41]	< 0.0001	2.14	[1.35–3.40]	< 0.01
Abdominal pain	0.33	[0.42–2.60]	0.30			
Dyspnea	2.20	[1.0–5.01]	0.06			

Ref. * Reference, ***aOR¶*** Adjusted Odds Ratio, ***n*** **=** 657^1^**:** corresponding to 657 FPEs

**Clinical symptoms**^**a**^**:** the following symptoms (Myalgia, Sore throat and Arthralgia) were not included in the analysis because they were missing in children under 5 years

**Headache**^**b**^**:** missing data for headache were excluded in the analysis

**Table 6:** factors associated with laboratory-confirmed influenza in patients >= 5 years, Dielmo and Ndiop cohort study, Senegal, 2013–2016 (*n* = 996^2^)

Variable	Univariate analysis			Multivariate analysis		
	OR	95% CI	***P*** value	aOR¶	95% CI	***P*** value
**Sex**						
Women	1.0	[0.73–1.30]	0.85			
**Clinical symptoms**						
Headache^1^	2.74	[1.16–6.50]	0.02			
Cough	6.26	[4.41–8.91]	< 0.0001	2.70	[1.94–3.72]	< 0.0001
Sore throat^1^	2.14	[1.50–3.10]	< 0.0001	1.75	[1.20–2.60]	< 0.01
Asthenia grade 0	Ref.*					
Asthenia grade 1	1.30	[0.52–3.20]	0.60	0.72	[0.36–1.50]	
Asthenia grade 2	1.41	[0.74–2.70]	0.30	1.15	[0.75–1.80]	
Asthenia grade 3	1.95	[1.10–3.52]	0.03	1.93	[1.30–2.85]	< 0.01
Anorexia	1.30	[0.95–1.72]	0.11			
Myalgia^1^	1.0	[0.75–1.32]	0.95			
Chills	1.0	[0.61–1.53]	0.87			
Nausea/Vomiting	0.55	[0.40–0.81]	< 0.01			
Sweats	0.72	[0.60–1.50]	0.90			
Arthralgia^1^	1.0	[0.70–1.51]	0.96			
Diarrhea	0.44	[0.21–0.95]	0.04			
Nasal discharge	7.38	[5.32–10.25]	< 0.0001	3.30	[2.44–4.43]	< 0.0001
Abdominal pain	0.50	[0.20–1.15]	0.31			
Dyspnea	3.70	[1.55–8.80]	< 0.01			

Ref. * Reference, **aOR¶:** Adjusted Odds Ratio, ***n*** **=** 996^2^**:** corresponding to 996 FPEs

Missing data for the following variables: Headache^1^, Myalgia^1^, Sore throat^1^ and Arthralgia^1^ were excluded in the analysis

In the multivariate model stratified by age group, cough and nasal discharge were significantly associated with influenza for all age group. Whereas, sore throat and grade 3 asthenia were being independently associated with influenza in the age group >= 5 years. (Tables 5 & 6).

### Performance of a modified diagnostic algorithm including “nasal discharge”

Table 7 shows that the addition of “nasal discharge” as a symptom to the WHO case definition (“fever > = 38 °C + cough + nasal discharge”) resulted in a decrease of sensitivity, regardless of age group, but equally in an increase of specificity. The decrease of sensitivity was significant (*p* < 0.05) for age groups ranging from 0- < 2 years to 15- < 50 years, but not among patients older than 50 years old (*p* = 0.16). The increase in specificity was significant (p < 0.05) for all age groups.

**Table 7:** Influenza Diagnostic Algorithm Performance Including the following Symptoms: Fever> = 38 °C + Cough + Nasal discharge

Case definition	Sensitivity	Specificity	PPV^*^	NPV ^**†**^	P-values
Fever >=38°+ Cough + Nasal discharge	**% (95% CI)**	**% (95% CI)**	**% (95% CI)**	**% (95% CI)**	
**All cases (n=1653)**	65.0 (60.0–70.0)	72.0 (69.5–74.4)	40.0 (36.1–40.0)	88.2 (86.2–90.1)	< 0.0001
**Age groups (years)**					
0 - < 2	59.1 (44.6–73.6)	60.0 (54.0–66.0)	20.0 (13.1–27.0)	89.5 (85.0–94.1)	< 0.01
2 - < 5	69.2 (59.0–79.4)	60.6 (55.0–66.4)	33.1 (26.0–40.3)	87.5 (83.0–92.2)	< 0.001
5 - < 15	69.0 (61.0–77.0)	77.5 (73.6–81.5)	48.0 (41.0–55.2)	89.3 (86.2–92.4)	< 0.001
15 - < 50	56.5 (46.0–67.0)	84.3 (80.1–88.6)	52.2 (42.0–62.4)	87.0 (82.5–90.5)	< 0.001
≥ 50	71.4 (52.1–91.0)	81.2 (70.1–92.2)	62.5 (43.1–82.0)	87.0 (77.2–97.0)	0.16

PPV^*^ Positive Predictive Value**,** ***NPV***^*†*^ Negative Predictive Value

The negative predictive values were all >= 87.0%. A nasal discharge together with fever/cough had the highest negative predictive value of 89.5% (IC95%: 85.0–94.1) but also the lowest positive predictive value of 20.0% (IC95%: 13.1–27.0) among the younger patients (Table 7).

## Discussion

This study evaluated both the performance of influenza case definitions and the clinical factors associated with the diagnosis of influenza, based on data from two sites within the national influenza surveillance network. Few studies have been conducted in a community context in rural areas in sub-Saharan Africa among patients presenting with fever, with or without respiratory signs (4). The strengths of this study are that: (i) influenza infection was confirmed by the RT-PCR technique – considered the gold standard; (ii) the database included a large pediatric population and has captured cases from several influenza seasons and different influenza sub-types.

During the 2013–2016 period, a significant proportion of the FPEs was confirmed by PCR to be positive for influenza PCR, with the occurrence of regular seasonal influenza epidemics between weeks 33 and 49 especially during rainy seasons. Since the beginning of circulation of the A *(H1N1)pdm* influenza strain in 2010 in Senegal*,* moderate peaks were observed between weeks 3 and 20 particularly in certain areas of the country. The study of environmental and climatic factors as well as the phylogenetic of the pandemic strain could lead to a better understanding of this apparent seasonality.

Our study found that patients more than 5 years were more often affected by febrile illnesses, with etiology being more often linked to respiratory tract pathogens other than influenza compared to the pediatric population [26]. In children under 5 years, the WHO, CDC and ECDC case definitions appear sensitive but not specific, especially in younger patients. The case definitions specificity improved in patients over 15 years old. This poor specificity of ILI case definitions in pediatric populations has been widely reported [13, 15–17, 27–29]. The variety of other potential co-infecting pathogens may have caused the lower specificity of case definitions in the younger age groups [16].

The relative performances of the WHO, CDC and ECDC ILI definitions in this study were similar to each other and to those obtained in other studies conducted in India (sensitivity 69–78% and specificity 43–65%) [13], and in Taiwan (sensitivity of 78% and specificity of 50% for the CDC case definition) in 2012 [30]. However, they differ from those found in a study conducted in France in 2016 (sensitivity between 84 and 98% and specificity between 4 and 27%) (*20*), and in another study carried out in Kenya in 2011 (sensitivity of 27% and specificity of 70%) [31]. These discrepancies can be explained by the different criteria for enrolling patients in studies, geographical influenza burden differences, and even the duration of the study.

Data collected between 2013 and 2016 in our patients’ cohort presenting with an FPE revealed, as previously reported in other studies, an increased risk of influenza infection that varies by age groups especially for those over 50 years [13, 20, 28, 29]. Previous studies have shown that people aged 50 and over with underlying health problems have a higher risk of influenza infection [32]. Other symptoms (sore throat, weakness) were also associated with an increased risk of influenza infection consistent with previous publications [4, 33]. However, in some studies, sore throat appeared to be associated with a decreased risk of influenza infection and supports the updated WHO definition from 2011 that removed sore throat from its definition [11, 20]. Cough was found as one of the most common symptoms associated with influenza in febrile patients of all age group, which is consistent with the results of other studies, and warranted the adoption of “fever ≥ 38° C and cough” as the WHO’s revised case definition of ILI in 2011 [13, 27–30, 34–36].

The other significant symptom related to positive diagnosis of influenza in all age group was nasal discharge, similar to Kathryn et al. findings [37]. We found the algorithm including “fever ≥ 38° C, cough and nasal discharge” less sensitive, especially in the younger patients, but more specific particularly in children. The corresponding negative predictive value was found to be higher in this age group compared to other age groups. This algorithm (“fever ≥ 38 ° C, cough and nasal discharge”) was reported in 2015 by Shah et al. as offering the best balance between sensitivity and specificity in children under 5 years of age [29].

The use of a very sensitive case definition requires substantial funds and resources for the processing of biological samples. In resource-limited settings, the addition of a symptom that enhances specificity criteria could help to reduce the number of false influenza positive samples collected and analyzed, which are otherwise costly and not informative about influenza viral types or subtypes in circulation. In our cohort, the addition of the “nasal discharge” to the WHO definition of ILI would reduce the number of false positives by 10% in Dielmo and Ndiop over the 2013–2016 period. This is important in light of the case definition encouraging the inclusion of other respiratory signs and symptoms in addition to a cough and fever.

### Limits and perspectives

This study has some limitations. The database used has a longitudinal character, implying the realization of repeated observations in several subjects, the same subject being able to have several FPEs. There is therefore a risk of correlation between these repeated observations in the same subject. The sampling and analysis were performed only in febrile patients, and it cannot be ruled out that the sensitivity was overestimated and the specificity underestimated. In addition, some of the symptoms or parameters included in the CDC and ECDC case definitions were not available in the database (malaise or shortness of breath), or did not meet the inclusion criteria (fever between 37.8 °C and 38 °C), which may have impacted the results by decreasing the performances of the different case definitions. Other limitations include missing or incomplete data among children under 5 years such as sore throat, arthralgia, myalgia and headache. The difficulty of eliciting a symptomatic history when the patient have limited language skills may contribute to this situation. This topic has been picked up in the literature and it is now established that this type of analysis, since it does not use all the information available in the database, induces a loss of power. In the case of a multivariate analysis, this type of analysis can also affect the variable selection process. Carry out a complete case analysis by including symptoms with missing data could highlight some of these symptoms as predictors of influenza infection.

The results of this study, in particular the performance of the “fever ≥ 38 ° C, cough and runny nose” algorithm for influenza surveillance, should now be tested across all 4S sentinel sites in order to confirm the results obtained in Dielmo and Ndiop.

## Conclusion

In summary, all three definitions studies (WHO, ECDC & CDC) have similar performance, even by age group. The revised WHO ILI definition could be chosen for surveillance purposes for its simplicity. Symptomatic predictors of influenza virus infection vary according the age group.

## Supplementary Information

**Additional file 1: Appendix 1.** Checklist criteria for 4S sentinel site selection

## Data Availability

The datasets used and/or analyzed during the current study are available from the corresponding author on reasonable request.

## Abbreviations

ILI: Influenza-like illness
WHO: World Health Organization
CDC: Center of Disease Control
ECDC: European Center for Disease Control
IPD: Institut Pasteur Dakar
NIC: National Influenza Center
FPE: Febrile Pathological Episode
PPV: Positive Predictive Value
NPV: Negative Predictive Value
PCR: Polymerase Chain Reaction
IQR: Interquartile range
SD: Standard deviation
