# Supplementary material for: Performance of case definitions and clinical predictors for influenza surveillance among patients followed in a rural cohort in Senegal
Source: BMC Infect Dis. 2021 Jan 7;21:31. doi: 10.1186/s12879-020-05724-x (PMC7790019; doi:10.1186/s12879-020-05724-x)
Supplement: Supplementary file 1 — Additional file 1: Appendix 1. Checklist criteria for 4S sentinel site selection [file 12879_2020_5724_MOESM1_ESM.docx]

**Appendix 1:** Checklist criteria for 4S sentinel site selection

| **Attribute** | **Criteria** |
| --- | --- |
| **Feasibility** | - Health center management agreeable to sentinel surveillance - At least two general practitioners available to screen patients - Local staff are motivated to participate in surveillance by adhering to case   definitions and collecting all necessary data and specimens   - Refrigerator for specimen storage available - Specimen courier service available - Computer and internet access available - Adéquater power supply for refrigerator - Back up generator available - Electronic or paper record for medical record system |
| **Representativeness** | - Community or district or province level hospital - Health center for all age groups - Health center for all socioeconomic level populations - All departments can participate in surveillance - General or speciality health center |
| **Disease burden calculation** | - Health center patient admissions per month - Patient home addresses available so that health center catchment population can be estimated |
